# Correlations between brain activity and components of motor learning in middle-aged adults: an fMRI study

**DOI:** 10.3389/fnhum.2013.00169

**Published:** 2013-05-06

**Authors:** Katie Wadden, Katlyn Brown, Rebecca Maletsky, Lara A. Boyd

**Affiliations:** ^1^Graduate Program in School of Rehabilitation Sciences, Faculty of Medicine, University of British ColumbiaVancouver, BC, Canada; ^2^Engineering ConsultantLawrence, KS, USA; ^3^Faculty of Medicine, Department of Physical Therapy, University of British ColumbiaVancouver, BC, Canada; ^4^Brain Research Centre, Faculty of Medicine, University of British ColumbiaVancouver, BC, Canada

**Keywords:** fMRI, motor sequence learning, temporal precision, implicit learning, correlation analysis, middle aged

## Abstract

Implicit learning may be shown by improvements in motor performance, which occur unconsciously with practice and are typically restricted to the task that was practiced. The purpose of this study was to examine behaviorally relevant brain activation associated with change in motor behavior during sequence-specific motor learning of a perceptuomotor continuous tracking (CT) task in middle-aged adults. To gain further insight into the neural structures associated with change in motor behavior, overall improvement in tracking (root mean square error; RMSE) was decomposed into two components—temporal precision and spatial accuracy. We hypothesized that individual differences in CT task performance would be evident in unique networks of brain activation that supported overall tracking behavior as well-temporal and spatial tracking accuracy. A group of middle-aged healthy individuals performed the CT task, which contains repeated and random segments for seven days. Functional magnetic resonance imaging (fMRI) data was collected on the first and seventh day while the participants performed the task. Subjects did not gain explicit awareness of the sequence. To assess behaviorally-relevant changes in the blood oxygenation level-dependent (BOLD) response associated with individual sequence-specific tracking performance, separate statistical images were created for each participant and weighted by the difference score between repeated and random performance for days 1 and 7. Given the similarity of performance for random and repeated sequences during early practice, there were no unique networks evident at day 1. On Day 7 the resultant group statistical fMRI image demonstrated a positive correlation between RMSE difference score and bilateral cerebellar activation (lobule VI). In addition, individuals who showed greater sequence-specific temporal precision demonstrated increased activation in the precentral gyrus, middle occipital gyrus, and putamen of the right hemisphere and the thalamus, cuneus, and cerebellum of the left hemisphere. Activation of this neural network further confirms its involvement in timing of movements as it has been previously associated with task performance when individuals are instructed to emphasize speed over accuracy. In the present study, behavioral performance was associated with neural correlates of individual variation in motor learning that characterized the ability to implicitly learn a sequence-specific CT task.

## Introduction

Implicit learning supporting the performance of complex motor sequences can be achieved in the absence of conscious knowledge of what was learned or that any learning took place (Frensch and Runger, 2003). Evidence for implicit motor learning comes from improved performance with skilled practice during sequential motor tasks (Rauch et al., 1995, 1997; Hazeltine et al., 1997; Willingham et al., 2002; Reiss et al., 2005). The majority of past work in this field employed the serial reaction time (SRT) task to examine implicit and explicit learning of sequences of movement (Nissen and Bullemer, 1987). In the SRT task, individuals follow visual cues to perform a series of motor responses; implicit learning is evidenced by faster reaction times for repeated as compared to random sequences (Schendan et al., 2003).

Despite extensive study, the neural correlates of implicit motor sequence learning remain somewhat elusive. A major reason for conflicting results in functional MRI (fMRI) studies of implicit learning is differences in experimental designs. For example, SRT task practice of shorter repeated sequences (less than 12 elements) can be acquired explicitly (Rauch et al., 1995), which alters patterns of brain activity (Doyon et al., 2002). In addition, the neural networks involved in practice phase acquisition performance differ greatly from those involved in implicit motor sequence learning when a relatively permanent change in behavior has occurred (Karni et al., 1995). Since the inception of the SRT task different variations have evolved, shifting dependent measures associated with learning from gross movement measures of the speed to more detailed analyses of kinematics, including measurements of temporal precision and spatial accuracy (Boyd and Winstein, 2004a). However, different patterns of brain activation are observed when participants learn inter-movement timing as compared to actual movements associated with a series of repeating motor responses (Gobel et al., 2011). Thus, in the present study we sought to eliminate many of the sources of variability outlined above by employing a continuous tracking (CT) task that does not easily stimulate explicit awareness of the repeated sequence (Boyd and Winstein, 2004b; Boyd et al., 2009), has no delays between stimuli for movement (Wulf and Schmidt, 1997), is not prone to ceiling effects despite extensive training (Wulf and Schmidt, 1997; Vidoni and Boyd, 2009) and can be dissociated into two components of performance, temporal precision, and spatial accuracy (Boyd and Winstein, 2004a) in order to differentiate unique networks of brain activation operating during motor sequence learning.

The ability to accurately index sequence-specific implicit motor learning relies heavily upon the design of the sequences to be learned (Wulf and Schmidt, 1997; Chambaron et al., 2006). Thus, in the present study we took additional methodological steps to equate tracking difficulty between the repeated and random sequences. We and others have noted variability in the magnitude of change in performance of the CT task, despite the provision of equal amounts of practice (Chambaron et al., 2006). Given this issue in the present study we undertook a new approach. Rather than considering group sequence-specific learning effects, we employed a subject-level analysis to ascertain how individual differences influenced both implicit learning of the repeated sequence and the neural networks that underpin these changes in motor behavior.

Thus, the purpose of the present study was to assess individual variation in behaviorally relevant brain activation with changes within the components of sequence-specific performance during a perceptuomotor task (ie., the CT task). We created sequence-specific weighted images to evaluate the correlations between increased brain activation during repeated sequence performance and the behavioral change score at a delayed retention test. We hypothesize that positive difference scores from repeated to random performance at retention will be associated with a distinct behavioral relevant fMRI activation changes in middle-aged adults following the completion of long-term practice of a CT task.

## Methods

### Participants

Ten right-handed healthy, middle-aged individuals participated (Mean = 64.7; *SD* = 8.5 years; six females). None presented with any evidence of dementia (26 or greater on the Mini-Mental State exam) (Folstein et al., 1975). Individuals were recruited from the university and local community. The rights of all participants were protected by the ethical review board at the University of British Columbia; each signed an approved institutional informed consent form prior to enrollment.

Participants were excluded if they had any history of major psychiatric diagnosis, substance abuse, or neurological disease or damage. Individuals who were taking any drug known to hamper motor learning or cortical plasticity (i.e., anticholinergics, GABAergics, NMDA receptor blockers as well as alpha and beta blockers) were not studied. Additional exclusion criteria were adopted for fMRI scanning (e.g., pregnancy, obesity, metallic objects in body, claustrophobia).

### Behavioral task

**Figure 1:**
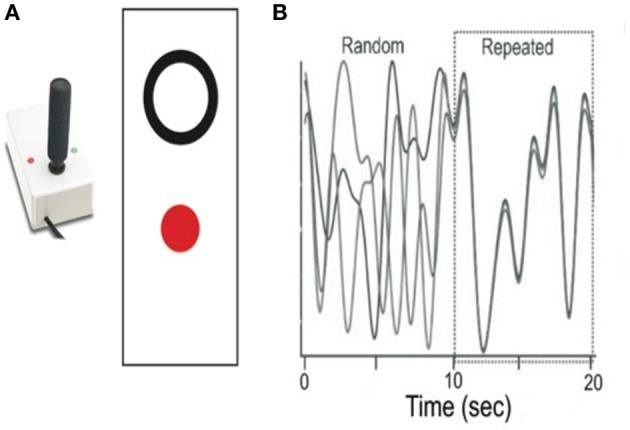
**Overview of experimental task and sample repeated and random segments. (A)** A non-ferrous joystick was used for tracking; individuals were asked to move the joystick to place the red dot in the white circle. **(B)** Sample repeated and random segments from the CT task.

A target circle outlined in white was visible on a black background as it moved up and down on a computer screen for a total of 20 s. Participants used their non-dominant arm to track the vertical path of the target with wrist movements that controlled a non-ferrous joystick (Current Designs^1^). Participants viewed the target on a 21″ monitor and made wrist motions from ~20° of ulnar deviation to 20° of radial deviation with the start position at 90°. Participants' movements were represented as a red filled circle. Each was instructed to “track the target as accurately as possible by trying to position the red filled circle inside the white open target” (Figure 1). Custom software developed on the LabVIEW platform was programmed to present all stimuli; joystick position was sampled at 50 Hz (v. 7.1, National Instruments, Inc.^2^ Co., Austin, TX, USA).

Unknown to the participants a predefined sequence of tracking pattern was embedded in the task for one segment of each trial, which remained identical across practice and retention. This pattern was constructed using the polynomial equation, as described by Wulf and Schmidt (1997) with the following general form:

$$f(x)=b_{o}+a_{1}\sin(x)+b_{1}\cos(x)+a_{2}\sin(2x)+b_{2}\cos(2x)+\;\cdots \;+a_{6}\sin(6x)+b_{6}\cos(6x)$$

The repeated sequence was constructed by using the same coefficients^3^ for every trial. The other segments of the tracking pattern were generated randomly using coefficients ranging from 10 to −10. This was calculated for each random segment so that the minimum and maximum of the segment were equidistant from the midline. The range of the random segments were restricted to a produce a range of motion within ±20° of movement. A different random array of segments was used for every trial; however, to ensure uniformity, the same random tracking patterns were practiced by all of the participants. During behavioral practice, random and repeated segments were linked such that participants were not aware when they were practicing each type. This was accomplished by adjusting the ends of each segment to cross the 0 point on the screen where they were linked one to another. In addition, the slope of the random segment was within 20% of the repeated segment at the point of transition.

Two screening methods were used in an attempt to equate the difficulty of the random and repeated segments. First, the range of motion of the random segment was calculated and the random segment was rejected if the range of motion was not within 5% of the range of motion of the repeated segment. Secondly, an average velocity criterion was developed using performance data from study participants for different random patterns. Based on the root mean squared error (RMSE) analysis, which reflects the overall movement error of tracking, the random patterns were ranked for each participant and then the ranking averaged across all subjects. This measure clearly identified segments that subjects consistently performed well or poorly. The average velocity for each random segment was calculated. There was a strong pattern showing segments with the lowest RMSE rankings (ranked as “easy”) also had low average velocity. The average velocity for the repeated cycle was calculated and compared with the values of the random segments. The value of the repeated segment average velocity was well above that of the “easy” random segments. Based on this analysis, an average velocity minimum was determined and segments with an average velocity lower than this value were eliminated from consideration. Both of these screening methods were employed as post processing. The random segments were analyzed; both for appropriate range of motion and average velocity, and segments not meeting these criterions were eliminated from further analysis.

The order of presentation of random and repeated segments were counterbalanced such that there was always an equal chance of performing either type of sequence first. The trajectories of the target and participants' movements did not leave a trail on the screen and thus, participants could not visualize the entire target pattern (Boyd and Winstein, 2004b). Participants practiced 50 trials (5 blocks; 10 trials/block) each behavioral day (i.e., days 2–6) under identical conditions. Participants were not explicitly informed of the existence of the repeating sequence but instructed daily to track the target as accurately as possible by controlling the position of the cursor with the joystick. This procedure was repeated for 5 days (250 trials total) to ensure adequate acquisition practice (Boyd and Winstein, 2001).

The same tracking task was used during fMRI acquisition (days 1 and 7). Participants' initial behavioral performance and blood oxygenation level-dependent (BOLD) response at baseline was assessed on Day 1. A separate retention test on Day 7 following the 5 days of practice was used to assess motor sequence learning and associated BOLD response. During these 2 days of fMRI acquisition, participant performed four separate runs of CT task performance that were comprised of 2 blocks presented in a block design (40 s rest/150 s stimulation/40 s rest/150 s stimulation/40 s rest) such that each block of stimulation consisted of either random or repeated sequences with the order of presentation within a scan counterbalanced across scans. During both behavioral practice and fMRI sessions the beginning and end of segments were not marked in any way, one segment simply ran into the next. The visual display was back projected (Panasonic LCD projector, model PT-L75OU) onto an opaque screen located above the participants' head and visible via a reflecting mirror located in the radio frequency head coil. Before the first fMRI session all participants were familiarized with the motor task. Prior to functional imaging, high-resolution 3D T1 images were collected for anatomic localization and co-registration (170 slices, 1.0 mm thickness, FOV = 256 mm).

### MRI data acquisition

Functional and anatomical imaging was performed at the UBC MRI Research Centre on a Philips Achieva 3.0 T whole body MRI scanner (Phillips Healthcare, Andover, MD) using a sensitivity encoding head coil (SENSE). Participants lay supine in the scanner with foam padding around their head to limit motion. The scanner was equipped with a three-axis local gradient radio frequency coil to collect whole brain fMRI (36 axial, 3 mm skip 1.0 mm slices). Functional imaging data were collected as axial echo-planar images, using a single-shot, blipped gradient-echo echo-planar pulse sequence (TE = 30 ms, TR = 2.0 s, 90° flip angle, FOV = 256 mm, 64 × 64). Four, 7 min runs of functional data were collected (210 images). Prior to functional imaging, high-resolution 3D T1 images were collected for anatomic localization and co-registration (170 slices, 1.0 mm thickness, FOV = 256 mm). Total scan time was ~45 min.

### Behavioral data analyses

Motor performance was evaluated across practice and retention in two ways. First we considered changes in RMSE, which is the average difference between the target pattern and participant movements and reflects overall tracking errors in the kinematic pattern^4^. This overall tracking error score was calculated separately for random and repeating segments on every tracking trial and averaged by block. RMSE for repeated sequences reflects sequence specific motor learning; changes in random sequence tracking error index non-specific improvements in motor control.

Next, we decomposed movement traces to determine spatial and temporal tracking accuracy. Spatial accuracy and the time lag between kinematic patterns and the target were deconstructed using a time series analysis (TSA). Only continuously overlapping sections of target and position data were correlated to avoid sample size biasing from different temporal shifts across trials and participants. Correlation coefficients (*R*^2^) reflect the spatial accuracy of tracking performance and the position of highest correlation was taken as the time lag as compared to target motion. Time lag of tracking represents the temporal distance from the target. The temporal offset was converted to milliseconds to characterize the temporal precision of tracking. Time lag scores that shifted from negative numbers to approach 0 (i.e., lower negative numbers indicate larger time lags while a 0 value represents no tracking time lag between participant and target) indicate improved motor performance. Finally, correcting for the contribution of time lag in the overall RMSE scores allowed us to determine spatial tracking error (Day and Marsden, 1982; Boyd and Winstein, 2004a,b).

Sequence-specific learning is indicated by superior performance (RMSE, temporal precision, and spatial accuracy) of the repeated than of the random sequence at the delayed retention test (Verwey et al., 2011).

### Explicit testing

Evaluation of explicit knowledge proceeded via testing for explicit recognition memory. We have employed this procedure in past studies, demonstrating its sensitivity to the acquisition of explicit awareness of recognition memory (Boyd and Winstein, 2004a; Boyd et al., 2009; Meehan et al., 2011). Ten-second sequences that only showed a target cursor were played on a 21″ computer screen. After viewing each sequence participants were asked to rate (yes/no) if they recognized the segment immediately after it finished playing (i.e., forced choice) (Boyd et al., 2009). In total, three true and seven novel or foil sequences were displayed. To be judged as having explicit knowledge participants had to correctly recognize 2 of 3 true sequences, and 4 of 7 false sequences as foils. These criteria for having acquired explicit knowledge of the repeated sequence are based on past published work (Boyd and Winstein, 2004a; Boyd et al., 2009; Meehan et al., 2011).

### fMRI data analysis

All data processing was performed using Analysis of Functional NeuroImages software (AFNI; Cox, 1996). Data from each participant and session was analyzed separately. Functional images were generated by condition (i.e., Rest, Random, and Repeated). The functional runs for each day were concatenated into a single file for data analysis. All functional images were spatially aligned to correct for head motion. No participant had head motion that exceeded 2 mm in any direction. A general liner model (GLM) was constructed using a deconvolution technique (Deconvolution Analysis FMRI Time Series,” http://afni.nimh.nih.gov/afni, D. Ward); individual time-course BOLD signal data were fit to the design matrix. In this fMRI block design, the model included the boxcar regressor of each condition of the design matrix (Repeated vs. Rest, Random vs. Rest) convolved with a hemodynamic response function (HDF). In addition, six predictors of no interest were included in the model to account for translational and rotational motion in the *x*, *y*, and *z* planes. Functional data were spatially smoothed using a 4 mm full-width-half-maximum Gaussian kernel. Anatomic images were registered in Talairach space and then co-registered with functional images (Talairach and Tournoux, 1988). The MRI Atlas of the Human Cerebellum (Schmahmann et al., 1999) was referenced to identify activation within the cerebellum.

To evaluate the relationship between sequence-specific motor learning and brain activation, a group analysis of individual statistical maps correlating brain activation during repeated sequence performance and behavioral difference score of overall improvement in tracking (RMSE), temporal precision, and spatial accuracy were created (Johansen-Berg et al., 2002). To ascertain this relationship, two separate analyses for Day 1 and retention (Day7) were performed to determine how the Repeated—Random BOLD contrast correlated with individual differences in performance and sequence-specific difference scores for each three measures (3 separate correlations analysis per day). Firstly, at the fMRI single-subject level, the Repeated—Random BOLD contrast from the GLM was produced creating a statistical image for each participant. Contrasts were performed separately on Day 1 and retention (Day 7) allowing for the direct evaluation of brain activation that was specific to repeated sequence learning. Activation observed on Day 1 is not associated with learning but rather performance measures. Each participant's statistical image for the Repeated – Random BOLD contrast was then multiplied by their corresponding difference score on Day 1 and retention [(mean repeated – mean random)] for RMSE, temporal precision, and spatial accuracy (Table 1) (Johansen-Berg et al., 2002). This created performance-weighted and sequence-specific weighted images for RMSE, mean temporal precision, and spatial accuracy difference scores on Day 1 and retention, respectively. RMSE and spatial accuracy difference scores signs were inverted during statistical weighting to assess positive correlations between increased sequence-specific performance and brain activation. At the group level, the single-subject contrasted images were summed and divided by the square root of the number of participants to create a correlated group statistical image (Johansen-Berg et al., 2002). Three separate performance-weighted and sequence-specific weighted images were generated for each day (Day 1 and 7) that represent a voxel-wise statistical image with a correlation at every voxel for brain activation and performance and sequence-specific difference score (Johansen-Berg et al., 2002). The initial performance-weighted and sequence-specific weighted image for RMSE learning analysis was thresholded at *p* < 0.005, with a spatial cluster threshold of 200 contiguous voxels; secondary analysis for the correlated temporal precision and spatial accuracy images were thresholded more conservatively at *p* < 0.0005, to minimize family-wise error and control for multiple comparisons. Day 1 was used to determine activation associated with performance of the repeated sequence, any overlapping activation observed between Day 1 and Day 7 was deemed performance-related activation rather than sequence-specific motor learning-related activation.

**Table 1:** **Participant characteristics and fMRI-weighted differences score at retention for performance during CT task inside the MR scanner**.

**Participant**	**Age**	**Sex**	**Overall movement error**	**Temporal precision**	**Spatial accuracy**
			**fMRI weighted difference score**		
1	54	F	0.702	−0.328	−0.534
2	64	F	0.084	0.116	0.201
3	72	F	0.123	−0.033	−0.141
4	67	F	−0.574	0.130	−0.342
5	60	F	−0.009	0.358	−0.422
6	51	M	−0.061	0.361	−0.206
7	63	M	−0.220	0.201	−0.268
8	68	M	−1.38	0.036	−0.899
9	80	F	−0.347	0.538	−0.676
10	69	M	−0.584	0.296	−0.655

### Statistical analysis

#### Behavioral data

Changes in tracking (overall movement error, temporal precision, and spatial accuracy) were analyzed across motor sequence practice sessions (Day 2–6) and in the MRI scanner (Day1 and 7). To evaluate the performance related changes in sequence specific learning separate repeated measures for Day (2–6) and Day (1–7) by Sequence (random, repeated) ANOVAs were conducted for overall movement error, temporal precision, and spatial accuracy of tracking change scores. Mauchly's test indicated that the assumption of sphericity had not been violated [χ^2^_(9)_ = 0.279, *p* > 0.05]. Outliers were deemed and removed if the changes in tracking were greater than two standards deviations of the group mean.

## Results

### Behavioral data

**Figure 2:**
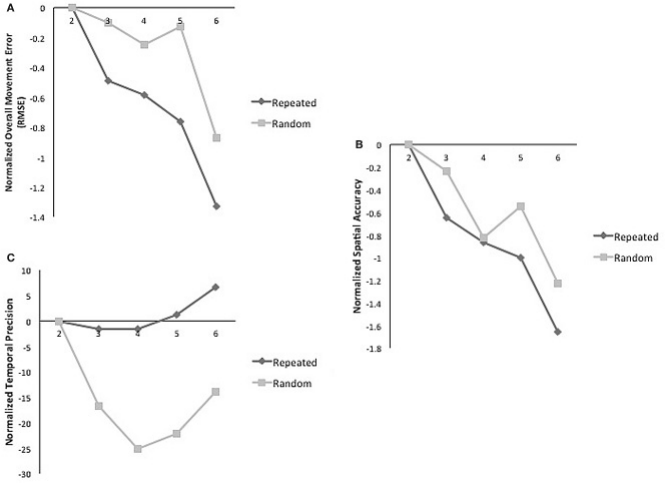
**(A)** Normalized mean RMSE for random and repeated sequences on practice days. Significant interaction of sequence and time [*F*_(1, 4)_ = 2.62; *p* = 0.05; η^2^ = 0.22; δ = 0.289]. **(B)** Normalized mean spatial accuracy for random and repeated sequences on practice days; main effect of time [*F*_(1, 4)_ = 6.01; *p* = 0.001; η^2^ = 0.40; δ = 0.97]. **(C)** Normalized mean temporal precision for random and repeated sequences on practice days. Significant interaction of sequence and time [*F*_(1, 4)_ = 2.836; *p* = 0.04; η^2^ = 0.706; δ = 0.262].

**Figure 3:**
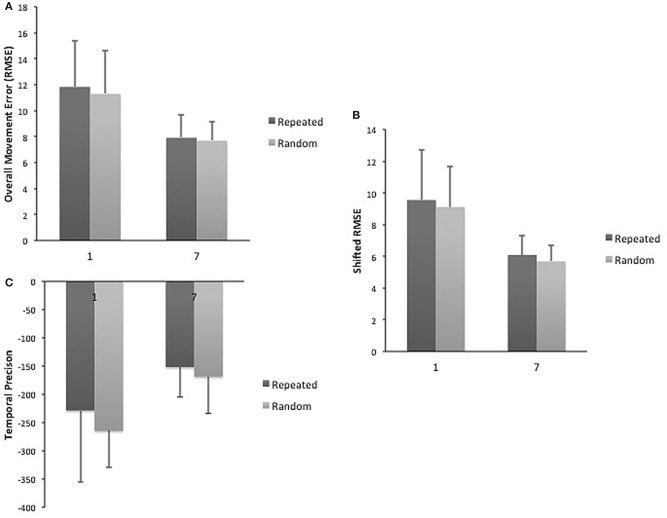
**(A)** Mean RMSE for repeated and random sequences on day 1 and 7. **(B)** Mean spatial accuracy for repeated and random sequences on day 1 and 7. **(C)** Mean temporal precision for repeated and random sequences on day 1 and 7.

A day by sequence interaction demonstrated that across practice days more improvement occurred for repeated sequence as compared to the random sequence for overall movement error (RMSE) (2–6) [*F*_(1, 4)_ = 2.61, *p* = 0.05; η^2^ = 0.226; δ = 0.675]. However, a main effect of day demonstrated that practice benefitted tracking performance for both types of sequences [*F*_(1, 4)_ = 4.19, *p* = 0.007; η^2^ = 0.32; δ = 0.884] (Figure 2A). When decomposed into the components of movement error, a significant main effect of day for spatial accuracy for both types of sequences [*F*_(1, 4)_ = 6.01; *p* = 0.001; η^2^ = 0.40; δ = 0.97] was observed (Figure 2B). Performance in temporal precision showed a significant interaction as greater improvements in this component were observed for repeated sequence tracking across practice [*F*_(1, 4)_ = 2.836; *p* = 0.04; η^2^ = 0.706; δ = 0.262] (Figure 2C). There was no significant difference between repeated and random sequences performance on day 1 or day 7 for any of the measures of tracking performance (Figure 3).

Participants failed to gain explicit knowledge as shown by their poor ability to correctly recognize the repeated sequences. Explicit recognition was at chance; only 55% of sequences were correctly identified.

### fMRI sequence-specific learning-related change

**Figure 4:**
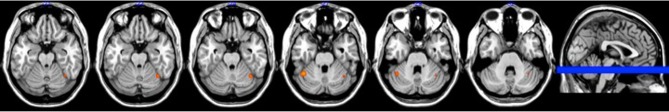
**Brain activation associated with overall movement error at retention (*p* < 0.005)**.

**Figure 5:**
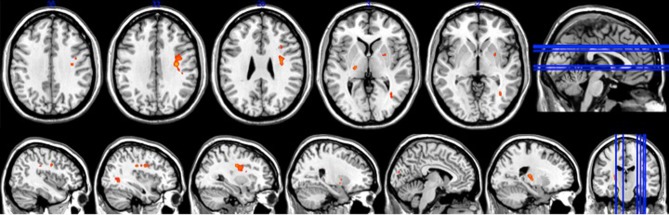
**Brain activation associated with temporal precision at retention (*p* < 0.0005)**.

There was no overlap between behaviorally correlated brain activation from Day 1 to retention, demonstrating the existence of a sequence-specific motor network. The correlational analysis for the positive differences scores at the retention test produced overall movement error (RMSE) and temporal precision sequence-specific weighted statistical images. Spatial accuracy did not show any positive correlations associated with increase brain activation at the retention test. The positive difference score in overall tracking performance was evident in three out of the 10 participants (Table 1). For these individuals, the RMSE sequence-specific weighted statistical image showed increased activity of the bilateral cerebellum (lobule VI) that correlated with positive difference scores at retention (Table 2; Figure 4). Decomposing overall movement error into its temporal and spatial components, identified only positive differences in temporal precision (time lag) during the repeated sequence with positive correlations in the precentral gyrus, middle occipital gyrus, and putamen of the right hemisphere and the thalamus, cuneus, and cerebellum of the left hemisphere (Table 3; Figure 5). These results were related to eight of the 10 subjects, as the positive difference scores in temporal precision were observed in eight of the 10 subjects (Table 1). There was no overlapping activation between Day 1 and Day 7, thus, the activation observed on Day 7 is behaviorally-related to sequence-specific learning.

**Table 2:** **Regional activation for correlational analysis for sequence-specific weighted image for overall movement error (root mean square error; RMSE) difference scores (mean repeated − mean random) at retention**.

**Brain region**	**Hemisphere**	**Cluster size**	***x***	***y***	***z***
**POSITIVE CORRELATIONS**			
Cerebellar lobule VI	R	313	37	−56	−25
Cerebellar lobule VI	L	306	41	52	−27

**Table 3:** **Regional activation for correlational analysis for sequence-specific weighted image for temporal precision difference score (mean repeated − mean random) at retention**.

**Brain region**	**Hemisphere**	**BA**	**Cluster size**	***x***	***y***	***z***
**POSITIVE CORRELATIONS**						
Precentral gyrus	R	6	1723	34	−8	30
Thalamus	L		724	−22	−21	8
Middle occipital gyrus	R	37	301	37	−61	2
Cuneus	L	18	285	−12	−86	16
Putamen	R		266	27	3	1
Cerebellar lobule VI	L		237	−35	69	−25

## Discussion

This study confirms past work showing that a broad network of brain activity supports sequence-specific learning of perceptuomotor tasks. Sequence-specific motor learning, as measured by the difference score in overall tracking error (RMSE) between repeated and random sequences at retention, was associated with increased activity in large bilateral clusters within the cerebellum (lobule VI). However, as this positive difference in behavior was only evident in three of the 10 subjects. Sequence-specific motor improvements were largely the result of improved (i.e., shorter) time lag of tracking (eight of the 10 subjects), denoted following the decomposition of overall movement error into the temporal and spatial components. We observed a relationship between improvements in temporal precision during sequence-specific motor learning and activation within a specific bihemispheric network of the brain—the precentral gyrus, middle occipital gyrus, putamen, thalamus, cuneus, and cerebellum. Limited literature exists on the long-term neural changes associated with improvements in the components motor learning in middle-aged adults, thus, the whole brain correlational analysis employed here provides a more comprehensive portrait of cortical reorganization associated with implicit motor sequence learning.

### Sequence-specific motor learning and associated brain activation

The sequence-specific weighted correlational analysis identified activation within both hemispheres of the cerebellum associated with sequence-specific motor learning as measured by changes in RMSE from repeated to random sequence performance. Activation associated with superior repeated motor learning was observed in bilateral lobule VI of the cerebellum. Past work suggested that during motor sequence learning, activation within areas of the cerebellum is dependent on the phase of learning (Doyon et al., 2002). The improved movement proficiency of the repeating pattern required bilateral activation of the cerebellum as a result of the kinetically demanding nature of the CT task. Other work supports our interpretation; for example during performance of an adapted SRT task that defines successful performance as acquisition of a rhythmic timing pattern during finger sequence key pressing rather than improvement in reaction time, increased activation of the lateral cerebellum during repeated sequence performance and decreased activation during random sequence performance are noted (Sakai et al., 2002). In the random condition the authors hypothesized that the cerebellum becomes disengaged with practice, as it is impossible to learn a temporal pattern (Sakai et al., 2002). Activation within the bilateral cerebellum cortex observed in present study demonstrates the importance of this region in the acquisition of a movement pattern with an emphasis on timing criterion.

### Behavioral changes

Practice-related changes were tracked across the five practice days outside the scanner. Consistent with previous findings, older adults showed repeated sequence-specific improvements during practice of the CT task (Howard and Howard, 1992, 1997; Boyd et al., 2007, 2009; Seidler et al., 2007). The CT task performed in the current study was a kinetically demanding task that allowed for the analysis of both spatial and temporal components of motor learning. When decomposed into the spatial and temporal components, spatial accuracy showed improvements for both sequences while temporal precision was superior for repeated compared to random sequence performance across practice. When the sequence-specific motor difference score (repeated minus random) was calculated at retention inside the scanner, an improvement in temporal precision was observed in eight of 10 participants, while sequence-specific motor learning for spatial accuracy was only observed in one of the participants. A dominant view in cognitive neuroscience suggests that aging has a negative effect on the speed of motor response times (Smith and Brewer, 1985; Starns and Ratcliff, 2010; Forstmann et al., 2011). The middle-aged adults in the present study did not appear to experience this negative consequence of age. Rather, they appeared to maintain the ability to increase the speed of tracking to improve motor performance. In the present study, learning the motor task did not require the middle-aged adults to make a decision; rather correctness of performance was based upon the instructional demands—“track the moving target as accurately as possible.” Given that CT task target moves at a fixed speed it appears that our participants chose to focus their efforts on staying as close to the target as possible which manifested itself as improved time lag of tracking.

### Temporal precision and associated brain activation

The effect of practice is related to changes in the movement structure of the motor sequence as learner's transition into the motor processing phase of learning (Park and Shea, 2005). Improved time lag during sequence-specific motor learning is indicative of increased motor processing efficiency. In the present study, individuals' ability to generate temporally proficient continuous movements during sequence-specific motor performance was associated with a network of brain regions. Several of the regions within this network, which were noted in the present study are well-known to be integral during motor sequence learning: premotor cortex, putamen, and the cerebellum (Doyon et al., 1998, 2002, 2003, 2009). In addition, these regions have been previously observed in tasks when individuals were instructed to emphasize speed over accuracy of performance (Van Veen et al., 2008). When emphasis is placed on speed, the premotor network provides top-down control, activating learned motor representations for response preparation (Van Veen et al., 2008). Change in activation of the premotor cortex has recently been correlated with learning scores during a visuomotor sequence-tracking task (Tomassini et al., 2011). It is possible that as sequence-specific motor learning was achieved, participants utilized this top-down control neural network to gain superior temporal acuity (Meehan et al., 2011). Consistent with previous evidence, the premotor cortex is involved in the shift from early visual-spatial processing, as seen in feedback-based control, to later motor processing, or feedforward-based control (Hikosaka et al., 1999). The neural network active in the present study was largely centered in the premotor area, which extended to the insula cortex. Activation of the insular cortex is associated with the involvement movement preparation and execution (Cross et al., 2007). In the present study increased activation of this area translated to decreased time lag as participants demonstrated superior motor planning as of the acquired kinetic movement pattern of the repeated sequence (Cross et al., 2007). The middle-aged adults who demonstrated increased activation of the premotor area, extending to the insula cortex, showed greater temporal precision resulting in overall lower tracking error.

The premotor regions with the thalamus and striatum form the cortico-striato-thalamo-cortical circuits (Middleton and Strick, 2002), and are known to actively communicate during speed emphasized tasks (Van Veen et al., 2008). While neither temporal nor spatial accuracy was given instructional favoritism, as participants were merely asked to follow the moving target, individuals who showed greater overall change in ability during the CT task appeared to prioritize temporal precision. The activation of the striatum may be reflective of a preference for timing accuracy over spatial acuity. The basal ganglia operates to gate information flow from frontal cortex to the motor system; when emphasis is placed on the speed of response the striatum increases activation as it receives increasing input from cortical sources (Forstmann et al., 2011). It is hypothesized that faster responses are associated with reciprocal communication from the cortex to the striatum. The striatum receives input from the cortex when the speed of performance is stressed; this enables a reduction in the inhibitory regulation the nuclei of the basal ganglia exert on the cortex leading to faster responses (Smith et al., 1998). In the present study, middle-aged individuals with higher activation levels of the putamen demonstrated greater temporal precision during sequence-specific motor learning at retention. Thus, this finding further supports the importance of premotor cortex and striatum activation in faster motor performance.

Feedforward tracts project from the cortex via pontine nuclei, decussate and terminate within the lobules of the cerebellum, forming the cerebo-ponto-cerebellar pathway (Salmi et al., 2010). This area has been linked structurally and functionally with areas within the motor and premotor cortex, having reciprocal projections with these areas of cortex (Kelly and Strick, 2003; Salmi et al., 2010). In agreement with the findings in the present study, activation with in the lobules VI of the cerebellum is associated with sensory-motor processes including temporally precise, visually guided, feedforward voluntary movements, and corrective development of motor sequence routines during the performance of the SRT task (Lehericy et al., 2005; Boyd et al., 2009). However, when individuals gain expertise or automaticity of movement, activation of the ipsilateral lobules of the cerebellum decreases (Jenkins et al., 1994; Doyon et al., 2002; Grafton et al., 2002). Our study suggests that individuals who performed with greater temporal precision activated the cerebellum to a greater extent than those who were not as proficient in tracking time lag. A possible explanation for the conflicting findings may lie in the nature of the CT task. The CT task relies heavily on visuospatial processing of a target more along a predefined spatial and temporal trajectory. Thus, continued activation of this area in accompaniment with premotor cortex and striatum might be important in feedforward control of a learned pattern of continuous movements at a predefined temporal trajectory.

## Conclusion

During 5-days of practice middle-aged adults showed overall improvement in movement error of the CT task and demonstrated sequence-specific motor learning as shown by superior repeated sequence performance. The importance of a subject-level analysis was fundamental in dissociating behaviorally-related changes of fMRI activation associated positive differences scores between repeated and random sequence performance at retention. The decomposition of overall tracking accuracy into the spatial and temporal components showed a behavior-related difference between repeated and random sequence performance. Sequence-specific motor learning was greatly reliant upon improvements in temporal precision and accompanied by activation of a specific neural network—premotor, thalamus, putamen, and cerebellum. Sequence-specific motor learning required participants to use top-down control as demonstrated by activation of the premotor area and the striatum. Activation of the premotor area shows participants were preparing for the upcoming learned movement pattern, whereas striatum activation supports the increased speed of movement. Thus, the observed brain areas associated with behavioral changes at retention provide insight into the neural correlates associated with different learning strategies during a CT task in middle-aged adults.

### Conflict of interest statement

The authors declare that the research was conducted in the absence of any commercial or financial relationships that could be construed as a potential conflict of interest.

## Footnotes

^1^Current Designs Inc., 3950 Haverford Ave., Philadelphia, PA 19104, USA.

^2^National Instruments Corporation, 11500 N Mopac Expressway, Austin, TX 78759-3504, USA.

^3^*b*_*o*_ = 4.37, *a*1 = −1.0, *b*1 = −4.6, *a*2 = −6.1, *b*2 = 9.6, *a*3 = −5.2, *b*3 = −7.1, *a*4 = 4.6, *b*4 = −4.5, *a*5 = −5.6, *b*5 = 3.6, *a*6 = −4.8, and *b*6 = 8.9.

^4^RMSE = ∑{(*x*_*i*_ − *T*_*i*_)^2^/*n*}^1/2^ *i* = 1 *x*_*i*_ = participant's position in degrees at time 1, *T*_*i*_ = target position at time 1, *n* = the number of samples for the participant's trajectory array.
